# Accuracy of urgency allocation in patients with shortness of breath calling out-of-hours primary care: a cross-sectional study

**DOI:** 10.1186/s12875-024-02347-y

**Published:** 2024-03-27

**Authors:** Michelle Spek, Roderick P. Venekamp, Esther de Groot, Geert-Jan Geersing, Daphne C. A. Erkelens, Maarten van Smeden, Anna S. M. Dobbe, Mathé Delissen, Frans H. Rutten, Dorien L. Zwart

**Affiliations:** 1 Department of General Practice & Nursing Science, Julius Centre for Health Sciences and Primary Care, University Medical Centre Utrecht Utrecht University, Utrecht, The Netherlands; 2 Department of Epidemiology, Julius Centre for Health Sciences and Primary Care, University Medical Centre Utrecht Utrecht University, Utrecht, The Netherlands

**Keywords:** Telephone triage, Netherlands triage standard, Out-of-hours primary care, Shortness of breath

## Abstract

**Background:**

In out-of-hours primary care (OHS-PC), semi-automatic decision support tools are often used during telephone triage. In the Netherlands, the Netherlands Triage Standard (NTS) is used. The NTS is mainly expert-based and evidence on the diagnostic accuracy of the NTS’ urgency allocation against clinically relevant outcomes for patients calling with shortness of breath (SOB) is lacking.

**Methods:**

We included data from adults (≥18 years) who contacted two large Dutch OHS-PC centres for SOB between 1 September 2020 and 31 August 2021 and whose follow-up data about final diagnosis could be retrieved from their own general practitioner (GP).

The diagnostic accuracy (sensitivity and specificity with corresponding 95% confidence intervals (CI)) of the NTS’ urgency levels (high (U1/U2) versus low (U3/U4/U5) and ‘final’ urgency levels (including overruling of the urgency by triage nurses or supervising general practitioners (GPs)) was determined with life-threatening events (LTEs) as the reference. LTEs included, amongst others, acute coronary syndrome, pulmonary embolism, acute heart failure and severe pneumonia.

**Results:**

Out of 2012 eligible triage calls, we could include 1833 adults with SOB who called the OHS-PC, mean age 53.3 (SD 21.5) years, 55.5% female, and 16.6% showed to have had a LTE. Most often severe COVID-19 infection (6.0%), acute heart failure (2.6%), severe COPD exacerbation (2.1%) or severe pneumonia (1.9%).

The NTS urgency level had a sensitivity of 0.56 (95% CI 0.50–0.61) and specificity of 0.61 (95% CI 0.58–0.63). Overruling of the NTS’ urgency allocation by triage nurses and/or supervising GPs did not impact sensitivity (0.56 vs. 0.54, *p* = 0.458) but slightly improved specificity (0.61 vs. 0.65, *p* < 0.001).

**Conclusions:**

The semi-automatic decision support tool NTS performs poorly with respect to safety (sensitivity) and efficiency (specificity) of urgency allocation in adults calling Dutch OHS-PC with SOB. There is room for improvement of telephone triage in patients calling OHS-PC with SOB.

**Trial registration:**

The Netherlands Trial Register, number: NL9682.

**Supplementary Information:**

The online version contains supplementary material available at 10.1186/s12875-024-02347-y.

## Background

Outside regular working hours, out-of-hours primary care (OHS-PC) provides urgent primary care to ensure 24/7 medical access. In the Netherlands, as in many other European countries, OHS-PC is organized in large scale cooperatives [1]. Under supervision of a general practitioner (GP), triage nurses assess by telephone the urgency of the patients’ health problem and decide whether the patient should be seen by a GP or by another medical professional, within what time frame, and what type of contact is needed (immediate ambulance, home visit, consultation with a GP or telephone advice) [2].

Semi-automatic decision support tools are often used during telephone triage [3, 4]. Albeit five-level triage systems are typically used to determine the required action and associated response time for a specific patient, different systems are used across healthcare settings [5–7]. Since 2011, the Netherlands Triage Standard (NTS) has been implemented in the Dutch out-of-hours primary care (OHS-PC) setting to assist nurses in the telephone triage process [1, 7]. The NTS is a semi-automatic decision support tool that requires the triage nurse to decide which of the 56 entrance complaints to choose from based on the symptoms presented by the caller. After the triage nurse completed approximately five questions, the NTS automatically generates an urgency level allocation with an associated (maximum) response time ranging from U1 to U5; U1 (immediate ambulance deployment), U2 (as quickly as possible, within 1 hour), U3 (within 3 hours), U4 (within 24 hours), U5 (telephone advice) [7]. The suggested urgency by the NTS can be overruled by the triage nurse, with or without consultation with the supervising GP [2, 6, 8]. The decision to overrule the NTS urgency to another urgency level if deemed appropriate is entirely at the discretion of the triage nurse (with or without consultation of the supervising GP).

The NTS is based on the Manchester triage system that was developed in and for emergency departments, but evidence on the performance of the NTS is scarce [6, 9]. Recently, the NTS has been validated against clinically relevant outcomes in patients calling OHS-PC with chest discomfort and in those with neurological deficit [8, 10–12]. These studies showed that NTS’ accuracy was moderate at best within these domains, emphasizing that there is room for improvement also in other domains, especially within those in which potentially life-threatening underlying diseases may present such as shortness of breath (SOB) [10, 11, 13, 14]. SOB is among the top five entrance complaints at OHS-PC and a prime reason for home visits by GPs [15]. Our aim was therefore to determine the accuracy of the NTS’ urgency allocation against life-threatening events (LTEs) in patients calling Dutch OHS-PC with SOB.

## Methods

### Study design and population

This study is part of the Opticall study, a cross-sectional study aimed at improving telephone triage of callers with SOB in Dutch OHS-PC. The rationale and design of this study is published elsewhere [16].

In short, we conducted a cross-sectional study including data of adult patients who called one out of two Dutch OHS-PC centres with SOB between 1 September 2020 and 31 August 2021 and in whom follow-up data about final diagnosis could be retrieved from the patients’ own GP’s electronic health record [17, 18]. We excluded children (< 18 years), patients whose triage conversation was not findable in the computer system, patients whose triage conversation was performed by an external organization, patients whose triage conversation was performed in a language other than Dutch or English, patients whose final urgency allocation was unknown, and patients whose telephone conversation was not for triage (e.g., a consultation with ambulance personnel).

### Data collection

Data was collected from both the OHS-PC and general practitioners. Patient characteristics, call characteristics, symptoms and urgency allocation were collected from re-listened call recordings and OHS-PC electronic health records (EHR). If a characteristic, sign or symptom was not mentioned during the telephone triage conversation, it was labelled as missing. These data from call recordings were linked to follow-up data about final diagnosis and hospitalization within 30 days of the index contact with the OHS-PC from the patients’ own primary care EHR.

### Outcome measures

Primary outcome was the diagnostic accuracy (sensitivity, specificity, positive predictive value, and negative predictive value) of the NTS urgency levels (high vs. low) in patients who call the OHS-PC service with SOB against LTEs as the reference.

The urgency allocation was stratified into high (U1 and U2) and low (U3, U4 and U5) urgency levels [7]. Life-threatening events (LTEs) justifying high urgency (U1-U2) included the following diagnoses: pulmonary embolism, acute coronary syndrome, acute heart failure, transient ischemic attack, stroke, sepsis, anaphylaxis, pneumothorax, subcutaneous emphysema, gastro-intestinal bleeding, Takotsubo cardiomyopathy, perforated diverticulitis, respiratory insufficiency and severe anaemia. The diagnosis COVID-19, pneumonia and asthma/COPD exacerbation were classified as either mild to moderate (in which U3-U5 was judged adequate) or severe (justifying U1-U2 and therefore also classified as LTE) with the latter defined as requiring hospital admission or supplemental oxygen administration at home within 24 hours of the OHS-PC index contact.

Secondary outcome was the diagnostic accuracy of the ‘final’ urgency levels, after potential overruling by triage nurses or supervising GPs.

### Data analysis

Baseline characteristics were described descriptively. Patient and call characteristics were compared between those with high and low NTS urgency allocation. The Pearson’s chi-square test or Fisher’s exact test (in case of groups with less than 10 people) was used to compare categorical variables. The independent sample T-test was used to compare continuous variables. We compared also with the Pearson’s chi-square test and independent sample T-test patient characteristics of eligible triage conversations included in the analysis against eligible conversations not included in the analysis.

Accuracy of telephone urgency allocation (high vs. low) was expressed as sensitivity, specificity, positive predictive value, and negative predictive value with corresponding 95% confidence intervals (CI) with LTE (yes vs. no) as the reference. McNemar’s Test was used to compare sensitivity and specificity of NTS urgency levels vs. ‘final’ urgency levels. In subgroup analyses, we stratified primary and secondary outcome analyses for gender and age categories < 40, 40–59, 60–79, > 80 years.

A *p*-value of < 0.05 was considered statistically significant. All data analyses were performed with SPSS statistics 26.0.

## Results

**Fig. 1:**
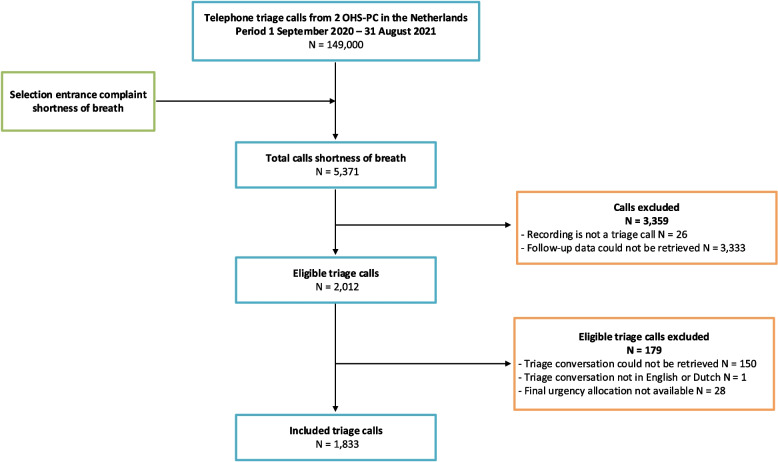
Flowchart of study population

A total of 2012 triage calls were eligible, of which we included 1833 triage calls. The mean age of the adult patients was 53.3 (SD 21.5) years and 55.5% were female. They called the OHS-PC with SOB between 1 September 2020 and 31 August 2021 (Fig. 1). Excluded patients did not differ from those included in the study in age (56.4 (SD 22.1) years vs. 53.3 (SD 21.5) years, *p* = 0.068) or gender (57.5% vs. 55.5% female, *p* = 0.606).

**Fig. 2:**
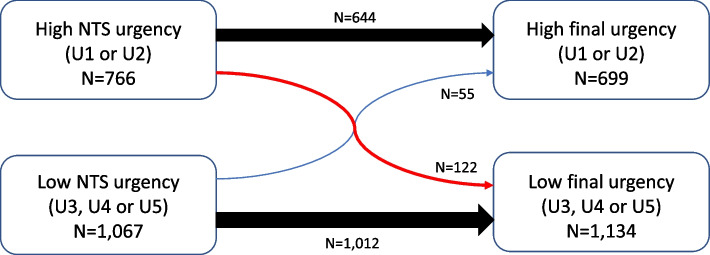
NTS urgency adjustments of 1833 callers who called the OHS-PC with SOB

Of the 1833 included patients, 766 (41.8%) received a high NTS urgency level (Fig. 2).

Baseline characteristics for callers with high and low NTS urgency allocation are shown in Table 1.

**Table 1:** Baseline characteristics of 1833 callers who called the OHS-PC with SOB, divided in those who received a high NTS urgency level allocation and low NTS urgency level allocation

	Total *n* = 1833	High NTS urgency level *n* = 766 (41.8%)	Low NTS urgency level *n* = 1067 (58.2%)	p-value
**Patient characteristics**				
Mean age in years (SD)	53.3 (21.5)	58.4 (20.7)	49.7 (21.4)	**< 0.001**
Male sex	815 (44.5%)	351 (45.8%)	464 (43.5%)	0.321
Female sex	1018 (55.5%)	415 (54.2%)	603 (56.5%)	0.321
**Call characteristics**				
Call duration in min:sec (SD) (*n* = 1818)*	12:53 (51:45)	14:43 (1:09:26)	11:33 (33:47)	0.245
Someone else called on behalf of patient (*n* = 1830)*	932 (50.9%)	476 (62.2%)	456 (42.8%)	**< 0.001**
GP participated in triage (*n* = 1831)*	870 (47.5%)	397 (51.9%)	473 (44.4%)	**0.001**
**Medical history**				
Cardiovascular disease (*n* = 912)*	306 (33.6%)	149 (40.3%)	157 (29.0%)	**< 0.001**
Respiratory disease (*n* = 1001)*	469 (46.9%)	223 (54.0%)	246 (41.8%)	**< 0.001**
Thrombo-embolic diseases (*n* = 651)*	22 (3.4%)	7 (2.9%)	15 (3.6%)	0.822
**Use of medication**				
Cardiovascular medication use	266 (14.5%)	120 (15.7%)	146 (13.7%)	0.235
Respiratory medication use	459 (25.0%)	211 (27.5%)	248 (23.2%)	**0.036**
Antithrombotic therapy	80 (4.4%)	38 (5.0%)	42 (3.9%)	0.290
**Symptoms mentioned during the call**				
Ankle oedema (*n* = 95)	54 (56.8%)	20 (57.1%)	34 (56.7%)	0.964
Autonomic nervous system related symptoms** (*n* = 1357)*	689 (50.8%)	340 (55.6%)	349 (46.8%)	**0.001**
Chest pain (*n* = 839)*	495 (59.0%)	221 (62.4%)	274 (56.5%)	0.084
Coughing (*n* = 1298)*	881 (67.9%)	346 (66.5%)	535 (68.8%)	0.400
Coughing blood (*n* = 1066)*	48 (4.5%)	20 (4.3%)	28 (4.6%)	0.832
Coughing sputum (*n* = 638)*	216 (33.9%)	84 (33.3%)	132 (34.2%)	0.822
Fever (*n* = 1252)	344 (27.5%)	151 (30.8%)	193 (25.4%)	**0.037**
Immobilisation (*n* = 84)*	58 (69.0%)	22 (81.5%)	36 (63.2%)	0.129
Malaise (*n* = 672)*	529 (78.7%)	209 (87.1%)	320 (74.1%)	**< 0.001**
Musculoskeletal pain (*n* = 209)*	168 (80.4%)	62 (87.3%)	106 (76.8%)	0.097
Palpitations (*n* = 154)*	99 (64.3%)	43 (72.9%)	56 (58.9%)	0.079
Shortness of breath (*n* = 1806)*	1747 (96.7%)	740 (97.9%)	1007 (95.9%)	**0.020**
Swollen calf (*n* = 41)*	5 (12.2%)	3 (20.0%)	2 (7.7%)	0.246
Tingling sensations (*n* = 102)*	68 (66.7%)	22 (66.7%)	46 (66.7%)	1.000
**Chest pain characteristics**				
Chest pain when breathing (*n* = 169)*	136 (80.5%)	57 (76.0%)	79 (84.0%)	0.190
Pain onset < 12 hours (*n* = 288)*	161 (55.9%)	81 (56.6%)	80 (55.2%)	0.802
Pain duration > 15 minutes (*n* = 248)*	237 (95.6%)	117 (95.9%)	120 (95.2%)	1.000
Posture-specific chest pain (*n* = 34)*	27 (79.4%)	12 (75.0%)	15 (83.3%)	0.681
Radiation of pain (*n* = 216)*	126 (58.3%)	74 (61.2%)	52 (54.7%)	0.342
Severe pain (score > 7 on VAS) (*n* = 98)*	26 (26.5%)	20 (30.3%)	6 (18.8%)	0.329
**Shortness of breath characteristics**				
SOB on exertion (*n* = 701)*	608 (86.7%)	239 (92.6%)	369 (83.3%)	**< 0.001**
SOB at rest (*n* = 1542)*	1484 (96.2%)	651 (98.6%)	833 (94.4%)	**< 0.001**
Stridor (*n* = 1135)*	37 (3.3%)	12 (2.7%)	25 (3.6%)	0.421
Unable to speak full sentences (*n* = 1507)*	222 (14.7%)	147 (24.3%)	75 (8.3%)	**< 0.001**
Wheezing (*n* = 1199)*	177 (14.8%)	79 (16.8%)	98 (13.5%)	0.114

*For these variables there were missing data

**Autonomic nervous system related symptoms consist of one or more of the following: nausea and/or vomiting, sweating, pallor/ashen skin, (near) collapse

*GP* general practitioner, *OHS-PC* out-of-hours primary care, *SOB* shortness of breath, *VAS* Visual Analogue Scale

Callers receiving high NTS urgency allocation were on average older than those receiving low urgency (58.4 (SD 20.7) vs. 49.7 (SD 21.4) years, *p* < 0.001), more often had someone else calling for them (62.2% vs. 42.8%, *p* < 0.001), and the supervising GP was more often involved in the triage process (51.9% vs. 44.4%, *p* = 0.001). They were also more likely to have a history of cardiovascular (40.3% vs. 29.0%, *p* < 0.001) and respiratory disease (54.0% vs. 41.8%, *p* < 0.001) than those receiving a low urgency. Regarding symptoms, callers receiving high NTS urgency were more likely to have autonomic nervous system related symptoms (55.6% vs. 46.8%, *p* = 0.001), fever (30.8% vs. 25.4%, *p* = 0.037) and malaise (87.1% vs. 74.1%, *p* < 0.001) than those receiving low urgency. Regarding the characteristics of SOB, those with SOB on exertion (92.6% vs. 83.3%, *p* < 0.001), SOB at rest (98.6% vs. 94.4%, p < 0.001), and/or unable to speak full sentences (24.3% vs. 8.3%, *p* < 0.001) were more likely to receive a high NTS urgency.

In 1280 (69.8%) callers, the initial NTS urgency level remained the ‘final’ urgency level. In the remaining 553 callers, the NTS urgency level was scaled up by the triage nurse in 228 (41.2%) and scaled down by the triage nurse in 325 callers (58.8%), both often after consultation with the supervising GP (See Table S1).

### Diagnoses

A complete overview of final diagnoses is provided in Table 2. In total, 16.6% had a LTE; most often severe COVID-19 infection (6.0%), acute heart failure (2.6%), severe exacerbation of COPD (2.1%) or severe pneumonia (1.9%).

**Table 2:** Diagnoses of 1833 callers to OHS-PC with shortness of breath, stratified by NTS urgency level

	Total n = 1833	High NTS urgency level n = 766 (41.8%)	Low NTS urgency level n = 1067 (58.2%)	p-value
**Life-threatening events**				
**Cardiovascular disorders**				
Acute coronary syndrome	13 (0.7%)	8 (1.0%)	5 (0.5%)	0.166
Acute heart failure	47 (2.6%)	30 (3.9%)	17 (1.6%)	**0.002**
**Respiratory tract disorders**				
Severe asthma exacerbation	11 (0.6%)	6 (0.8%)	5 (0.5%)	0.542
Severe COPD exacerbation	38 (2.1%)	26 (3.4%)	12 (1.1%)	**< 0.001**
Severe COVID-19 infection	110 (6.0%)	56 (7.3%)	54 (5.1%)	**0.045**
Severe pneumonia	34 (1.9%)	23 (3.0%)	11 (1.0%)	**0.002**
**Other disorders**				
Anaphylaxis	14 (0.8%)	1 (0.1%)	13 (1.2%)	**0.011**
Pulmonary embolism	16 (0.9%)	6 (0.8%)	10 (0.9%)	0.804
Sepsis	10 (0.5%)	7 (0.9%)	3 (0.3%)	0.070
Other life-threatening events (LTEs)*	12 (0.7%)	7 (0.9%)	5 (0.5%)	0.256
**Non-urgent disorders**				
**Cardiovascular disorders**				
Stable heart failure	39 (2.1%)	14 (1.8%)	25 (2.3%)	0.451
**Respiratory tract disorders**				
Mild or moderate asthma exacerbation	117 (6.4%)	39 (5.1%)	78 (7.3%)	0.055
Mild or moderate COPD exacerbation	94 (5.1%)	57 (7.4%)	37 (3.5%)	**< 0.001**
Mild or moderate COVID-19 infection**	387 (21.1%)	141 (18.4%)	246 (23.1%)	**0.016**
Mild or moderate pneumonia	80 (4.4%)	37 (4.8%)	43 (4.0%)	0.408
Upper respiratory tract infection	103 (5.6%)	26 (3.4%)	77 (7.2%)	**< 0.001**
**Other disorders**				
Hyperventilation/anxiety/stress	136 (7.4%)	41 (5.4%)	95 (8.9%)	**0.004**
Shortness of breath due to (existing) cancer	34 (1.9%)	19 (2.5%)	15 (1.4%)	0.093
Unspecified chest pain***	85 (4.6%)	43 (5.6%)	42 (3.9%)	0.092
Unspecified shortness of breath***	208 (11.3%)	63 (8.2%)	145 (13.6%)	**< 0.001**
Other non-urgent disorders****	245 (13.4%)	116 (15.1%)	129 (12.1%)	0.058

*LTE* life-threatening event, *OHS-PC* out-of-hours primary care

*transient ischaemic attack, stroke, pneumothorax, subcutaneous emphysema, gastro-intestinal bleeding, Takotsubo cardiomyopathy, perforated diverticulitis, respiratory insufficiency due to reduced consciousness, severe anaemia

**Proven (most cases) and suspected COVID-19 infections

**Cardiac pathology unlikely after cardiologist’s or GP’s diagnostic work-up, including those with musculoskeletal chest pain

***Cardiac or pulmonary pathology unlikely after cardiologist’s, pulmonologists, or GP’s diagnostic work-up

****Amongst others: atrial fibrillation or atrial flutter, gastro-oesophageal reflux, costal contusion/fracture, bronchitis or bronchial hyperreactivity, shortness of breath due to terminal phase, hay fever

### Urgency levels and LTE

Of those who showed to have had a LTE, 170 (55.7%) received a high NTS urgency level. The remaining 135 callers with a LTE mostly received a U3 urgency level (71.1%), followed by a U5 urgency level (16.3%) and U4 urgency level (12.6%) (See Tables S2 and S3). Of those who showed not to have had a LTE, 39.0% received a high NTS urgency level.

Of those who showed to have had a LTE, 165 callers (54.1%) received a high ‘final’ urgency level. The remaining 140 callers with a LTE mostly received a U3 urgency (80.0%), followed by a U4 urgency (10.7%) and U5 urgency (9.3%) (See Tables S2 and S3). Of those who showed not to have had a LTE, 34.9% received a high ‘final’ urgency level.

### Accuracy of NTS and ‘final’ urgency levels

Considering a high urgency allocation correct for those with a LTE and a low urgency allocation for those without a LTE, the NTS urgency allocation had a sensitivity of 0.56 (95% CI 0.50–0.61), a specificity of 0.61 (95% CI 0.58–0.63), a positive predictive value of 0.22 (95% CI 0.19–0.25) and a negative predictive value of 0.87 (95% CI 0.85–0.89).

The ‘final’ urgency level had a sensitivity of 0.54 (95% CI 0.48–0.60) and specificity of 0.64 (95% CI 0.63–0.67), a positive predictive value of 0.24 (95% CI 0.21–0.27) and a negative predictive value of 0.88 (95% CI 0.86–0.89) (See Table 3).

**Table 3:** Accuracy of NTS urgency level and ‘final’ urgency allocation for detecting LTE (prevalence of 16.6%) of 1833 callers who called the OHS-PC with SOB

	NTS urgency allocation (95% CI)	‘Final’ urgency allocation (95% CI)
**Sensitivity**	0.56 (0.50–0.61)	0.54 (0.48–0.60)
**Specificity**	0.61 (0.58–0.63)	0.65 (0.63–0.67)
**Positive predictive value**	0.22 (0.19–0.25)	0.24 (0.21–0.27)
**Negative predictive value**	0.87 (0.85–0.89)	0.88 (0.86–0.89)

*LTE* life-threatening events, *NTS* Netherlands triage standard, *OHS-PC* out-of-hours primary care, SOB: shortness of breath

Stratified analyses for gender yielded comparable results. Diagnostic accuracy in callers younger than 40 years had a higher specificity compared with other age categories (40–59, 60–79, > 80 years), while sensitivity was similar across all age categories (See Tables S4-S9). With an increase in prevalence of LTE with age, the positive predictive value was higher and the negative predictive value lower in older patients.

## Discussion

Among adults with SOB who called the OHS-PC in the Netherlands between September 2020 and August 2021, 16.6% had a LTE, including 6.0% with a severe COVID-19 infection. The semi-automatic decision support tool (NTS) used to help triage callers to the OHS-PC performed poorly with respect to safety (sensitivity: 0.56, 95% CI 0.50–0.61) and efficiency (specificity: 0.61, 95% CI 0.58–0.63). Overruling of the NTS’ urgency allocation by triage nurses and/or supervising GPs did not impact sensitivity (0.56 vs. 0.54, p = 0.458) and only slightly improved specificity (0.61 vs. 0.65, p < 0.001). The fact that the prevalence is lower, and specificity is slightly higher in younger callers is not surprising because at a lower prevalence, a test with high specificity usually identifies healthy individuals better; the likelihood of false-positive results is lower when only a small number of patients actually have a LTE. However, the consequences of missing LTEs are even more relevant in younger people because they have better chances of good long-term outcomes if LTEs are detected early. This might affect the weighing of sensitivity versus specificity depending on the age of an individual.

Looking at the positive predictive values, we see that for a caller with a high urgency, the probability of having a LTE increases only slightly from the prior probability of 16.6 to 22% (NTS urgency) or 24% (‘final’ urgency). On the other hand, a caller with low urgency still has a 13% (NTS urgency) or 12% (‘final’ urgency) chance of having a LTE.

### Comparison to literature

A study among Belgian regular GP practices showed that LTEs among patients with SOB were rare, and immediate hospitalization was needed in 4.4% of patients [19]. The incidence of LTEs is substantially lower than in our study (LTE occurred in 16.6%). Reasons could be that we performed our study during the COVID-19 pandemic (6.0% had severe COVID-19) and that callers with SOB who contact OHS-PC in general have more often severe underlying conditions than those contacting regular GP care during office hours.

The lack of studies focussing on telephone triage in patients with SOB hampers adequate comparison of our study findings to the wider body of literature.. Studies on the Manchester triage system, which is used in most European countries to triage patients in emergency departments, show conflicting results [20–24]. In patients with SOB, the Manchester triage system had a sensitivity of 0.76 and specificity of 0.66 with seven-day mortality as the outcome that occurred in 3.6% of the patients [21]. Higher sensitivity and specificity with the Manchester triage system compared to the NTS is driven by the setting (emergency department with high risk of LTE versus general practice with low risk of LTE), and more useful determinants because the MTS also includes items for which face-to-face consultation is needed [25, 26]. Telephone triage as with the NTS is based on the items mentioned during the call, and these need to be weighted in a short time window [27, 28]. Finally, a difference in the outcome hampers comparison (7-day mortality versus LTEs). Discriminating LTEs that result in death within 7 days from non-LTEs or LTEs with a more beneficial prognosis is easier than discriminating LTEs from non-LTEs.

While several studies, including a systematic review and a meta-analysis, were performed on diagnostic accuracy of the Manchester triage system with mortality or a clinical diagnosis as the reference, similar studies investigating diagnostic accuracy of the NTS or other semi-automatic triage tools in primary care are scarce and limited to patients with chest discomfort and neurologic deficit [8, 10–12]. These studies also performed in Dutch primary care reported also a moderate performance of the NTS-system; for LTEs in those with acute chest discomfort a sensitivity of 0.73 and specificity of 0.43, and for LTEs in those with neurologic deficit symptoms a sensitivity of 0.72 and specificity of 0.48 [10, 11]. However, overruling the NTS system by triage nurses and/or supervising GPs made the triage safer (sensitivity of 0.86 for both domains) while it remained inefficient (specificity of 0.34 and 0.38 for both domains, respectively). This contrasts with our study of patients with SOB in which we did not find a difference between the sensitivity of the NTS and the ‘final’ urgency level, and only a marginal better specificity of the ‘final’ urgency. This difference is possibly caused by the fact that calamities at OHS-PC are most often caused by missing myocardial infarction and acute cardiac death, so triage of a patient with chest pain may be more stressful for triage nurses and therefore they are possibly more likely to overrule the NTS system [10, 29]. For neurological deficit, triage nurses presumably know that the prevalence of a serious condition is higher so here too they may overrule to a higher urgency more often [11].

Our results are comparable with another study from our group in which we assessed the impact of the COVID-19 pandemic on telephone triage in callers with SOB and chest discomfort during the first wave of the COVID-19 pandemic and a pre-pandemic period [30]. Among those with SOB, a sensitivity of 0.64 (pre-pandemic) and 0.60 (COVID-19 pandemic) and a specificity of 0.56 (pre-pandemic) and 0.64 (COVID-19 pandemic).

The high negative predictive value found in our study is related to the relative low prevalence of 16.6%; in case of a low urgency level allocation, there is indeed a 83.4% chance that this patient does not have a LTE based on prevalence only. The low positive predictive value found in our study indicates a safe way of triaging callers with SOB who contact OHS-PC. This is consistent with a questionnaire among Dutch GPs on the applicability of the NTS and the overall quality of triage with the NTS in OHS-PC [31]. They subjectively felt that the NTS was too defensive [31]. Furthermore, since the introduction of the NTS in 2011, there has been an increase in high urgency allocations, which indeed indicates that NTS is more defensive than the triage nurse without a decision support tool as was the case before the introduction of the NTS [1, 31, 32]. On the other hand, this safe way of triaging these patients is not surprising because the acceptable missing rates for LTEs that might present with SOB are really low; less than 1% for acute coronary syndrome according to cardiologists and 3% for pulmonary embolism according to specialists involved in the diagnostic process and treatment of these patients [33, 34]. We should, however, realise that this safe way of triaging results in ‘overtriage’ and thus disrupts the acute care chain. This is undesirable because it may cause delays for patients who truly need urgent care, and in addition, it places an undesirable burden on health care resources, imposes unnecessary health care costs, may cause iatrogenic damage to the patient and, finally, is associated with a potentially preventable climate impact of healthcare use [35, 36].

### Strengths and limitations

We were in the unique position to evaluate real-life recordings of the initial contact of callers with SOB. The very first verbal symptom presentation was recorded, and these tape recordings were scored without knowledge of the final diagnosis, that is, without hindsight bias. Furthermore, this study includes a large population without strict exclusion criteria, resulting in a representative real-life study population. In addition, we were able to link data from callers to the OHS-PC with follow-up data from the patient’s own GP, including specialist letters if the patient was referred, for a reliable determination of the final diagnosis up to 30 days after the index contact to OHS-PC.

A limitation is that we selected callers where the entrance complaint SOB was chosen. Before choosing a specific entrance complaint, a triage nurse must determine whether a patient is ABCD stable or not. This means that callers with severe SOB who are ABCD unstable because of B(reathing) problems were not included in our study. With these callers, however, it will be clear that an ambulance is needed, so problems in triage will occur primarily with those with the entrance complaint SOB who are ABCD stable. However, if a triage nurse incorrectly identifies callers with a B(reathing) problem as ABCD stable and then chooses the entrance complaint SOB, these patients will be in our sample. Moreover, with nearly all patients in our study sample reporting SOB during the triage call, our sample resembles those experiencing SOB.

Our study period included the COVID-19 pandemic, which might have influenced our results and particularly the prevalence of LTEs as 6.0% of patients included in our study had a severe COVID-19 infection. Moreover, COVID-19 has been associated with a higher prevalence of LTEs, notably pulmonary embolism and acute coronary syndrome [37–39]. Importantly, however, there is no evidence of a difference in symptom presentation of LTEs in patients with or without concurrent COVID-19 infection. The mimicking symptomatology regarding SOB and chest discomfort may hamper triage between patients with mild to moderate COVID-19 and patients with LTE [40, 41]. Importantly, however, in another study, we could show that the sensitivity and specificity of the ‘final’ urgency allocation in patients with SOB was similar during the first COVID-19 wave and in the pre-pandemic period, indicating that the impact of COVID-19 – different than expected – on diagnostic accuracy in the domain of SOB in the OHS-PC setting was limited [30].

As per routine practice, not all of the included patients were transferred to the hospital for further diagnostic assessment. This may have led to some cases having initially incorrectly received an alternative diagnosis than LTE. To reduce such misclassification as much possible, we collected data about the final diagnosis from the patient’s primary care EHR up to 30 days after the index contact at the OHS-PC. It is therefore unlikely that any misclassification of LTEs has substantially influenced our main findings.

Another limitation was that we had to exclude 8.9% of eligible triage conversations. We could, however, show that patient characteristics did not differ between those with and without a final diagnosis based on follow-up information. Thus, this selection did not cause selection bias, the more so because the eligibility of triage conversations seems not to be associated with the medical outcome of individual callers.

### Implications for future research

Optimisation of both safety (avoiding ‘undertriage’) and efficiency (avoiding ‘overtriage’) of telephone triage is needed. This could probably be achieved by developing and validating a prediction model for LTEs among callers to OHS-PC with SOB [16]. If proven effective, such a model could be incorporated in the decision support tool and thus optimise the triage. Potentially other semi-automatic decision support tools for telephone triage used in other healthcare settings using OHS-PC could adept these changes, e.g. Scandinavian countries, Germany and the United Kingdom [21, 42].

## Conclusions

The semi-automatic decision support tool used to triage adults calling Dutch OHS-PC with SOB performs poorly with respect to safety (sensitivity) and efficiency (specificity). There is room for improvement of telephone triage in patients calling OHS-PC with SOB.

### Supplementary Information

**Supplementary material 1.**

## Data Availability

The data can be made available for researchers whose proposed use of the data has been approved at request of the corresponding author, with a signed data access agreement.

## Abbreviations

CI: confidence interval
EHR: electronic health records
GP: general practitioner
LTE: life-threatening event
NTS: Netherlands Triage Standard
OHS-PC: out-of-hours primary care
SD: standard deviation
SOB: shortness of breath
VAS: Visual Analogue Scale
