# Supplementary material for: Accuracy of urgency allocation in patients with shortness of breath calling out-of-hours primary care: a cross-sectional study
Source: BMC Prim Care. 2024 Mar 27;25:101. doi: 10.1186/s12875-024-02347-y (PMC10967202; doi:10.1186/s12875-024-02347-y)
Supplement: Supplementary file 1 — Supplementary material 1. [file 12875_2024_2347_MOESM1_ESM.docx]

# Supplementary file

**Table S1. NTS urgency level and ‘final’ urgency level allocation of 1,833 callers to OHS-PC with shortness of breath.**

|  | **Final U1** | **Final U2** | **Final U3** | **Final U4** | **Final U5** | **Total** |
| --- | --- | --- | --- | --- | --- | --- |
| **NTS U1** | 67 | 56 | 8 | 3 | 5 | 139 |
| **NTS U2** | 12 | 509 | 45 | 36 | 25 | 627 |
| **NTS U3** | 3 | 26 | 405 | 61 | 52 | 547 |
| **NTS U4** | 0 | 11 | 57 | 123 | 34 | 225 |
| **NTS U5** | 0 | 15 | 74 | 30 | 176 | 295 |
| **Total** | 82 | 617 | 589 | 253 | 292 | 1,833 |
| NTS: Netherlands triage standard, OHS-PC: out-of-hours primary care, SOB: shortness of breath  Black: the NTS urgency and ‘final’ urgency were the same  Blue: the user (triage nurse and/or supervising GP) has scaled up the urgency  Red: the user (triage nurse and/or supervising GP) has scaled down the urgency | | | | | | |

**Table S2. NTS urgency level and ‘final’ urgency allocation of 305 callers to OHS-PC with LTE.**

|  | **Final U1** | **Final U2** | **Final U3** | **Final U4** | **Final U5** | **Total** |
| --- | --- | --- | --- | --- | --- | --- |
| **NTS U1** | 16 | 9 | 1 | 0 | 0 | 26 |
| **NTS U2** | 5 | 123 | 9 | 3 | 4 | 144 |
| **NTS U3** | 1 | 7 | 85 | 2 | 1 | 96 |
| **NTS U4** | 0 | 2 | 7 | 8 | 0 | 17 |
| **NTS U5** | 0 | 2 | 10 | 2 | 8 | 22 |
| **Total** | 22 | 143 | 112 | 15 | 13 | 305 |
| NTS: Netherlands triage standard, OHS-PC: out-of-hours primary care, SOB: shortness of breath  Black: the NTS urgency and ‘final’ urgency were the same  Blue: the triage nurse and/or supervising GP has scaled up the urgency  Red: the triage nurse and/or supervising GP has scaled down the urgency | | | | | | |

**Table S3. Association between high urgency level allocation or low urgency level allocation and final diagnosis life-threatening disease of 1,833 callers with shortness of breath.**

| **NTS urgency level** | **LTE**  **n=305 (16.6%)** | **No LTE**  **n=1,528 (83.4%)** |
| --- | --- | --- |
| High NTS urgency level | 170 (55.7%) | 596 (39.0%) |
| Low NTS urgency level | 135 (44.3%) | 932 (61.0%) |
| **‘Final’ urgency level** | **LTE**  **n=305 (16.6%)** | **No LTE**  **n=1,528 (83.4%)** |
| High ‘final’ urgency level | 165 (54.1%) | 534 (34.9%) |
| Low ‘final’ urgency level | 140 (45.9%) | 994 (65.1%) |
| LTE: life-threatening event, OHS-PC: out-of-hours primary care | | |

**Table S4. Accuracy of NTS urgency level and ‘final’ urgency allocation for detecting LTE (prevalence of 18.8%) of 815 males who called the OHS-PC with SOB.**

|  | **NTS urgency allocation (95% CI)** | **‘Final’ urgency allocation (95% CI)** |
| --- | --- | --- |
| **Sensitivity** | 0.56 (0.47-0.64) | 0.52 (0.43-0.60) |
| **Specificity** | 0.60 (0.56-0.64) | 0.64 (0.60-0.68) |
| **Positive predictive value** | 0.24 (0.20-0.29) | 0.25 (0.20-0.30) |
| **Negative predictive value** | 0.85 (0.82-0.88) | 0.85 (0.82-0.88) |
| LTE: life-threatening event, NTS: Netherlands Triage Standard, OHS-PC: out-of-hours primary care, SOB: shortness of breath | | |

**Table S5. Accuracy of NTS urgency level and ‘final’ urgency allocation for detecting LTE (prevalence of 14.9%) of 1,018 females who called the OHS-PC with SOB.**

|  | **NTS urgency allocation (95% CI)** | **‘Final’ urgency allocation (95% CI)** |
| --- | --- | --- |
| **Sensitivity** | 0.56 (0.48-0.64) | 0.57 (0.48-0.65) |
| **Specificity** | 0.62 (0.59-0.65) | 0.66 (0.48-0.65) |
| **Positive predictive value** | 0.20 (0.17-0.25) | 0.22 (0.18-0.27) |
| **Negative predictive value** | 0.89 (0.86-0.91) | 0.90 (0.87-0.92) |
| LTE: life-threatening event, NTS: Netherlands Triage Standard, OHS-PC: out-of-hours primary care, SOB: shortness of breath | | |

**Table S6. Accuracy of NTS urgency level and ‘final’ urgency allocation for detecting LTE (prevalence of 6.2%) of 583 callers younger than 40 years who called the OHS-PC with SOB.**

|  | **NTS urgency allocation (95% CI)** | **‘Final’ urgency allocation (95% CI)** |
| --- | --- | --- |
| **Sensitivity** | 0.50 (0.33-0.67) | 0.47 (0.31-0.64) |
| **Specificity** | 0.73 (0.69-0.77) | 0.77 (0.73-0.80) |
| **Positive predictive value** | 0.11 (0.07-0.17) | 0.12 (0.07-0.18) |
| **Negative predictive value** | 0.96 (0.93-0.97) | 0.96 (0.93-0.97) |
| LTE: life-threatening event, NTS: Netherlands Triage Standard, OHS-PC: out-of-hours primary care, SOB: shortness of breath | | |

**Table S7. Accuracy of NTS urgency level and ‘final’ urgency allocation for detecting LTE (prevalence of 13.5%) of 483 callers aged 40-59 years who called the OHS-PC with SOB.**

|  | **NTS urgency allocation (95% CI)** | **‘Final’ urgency allocation (95% CI)** |
| --- | --- | --- |
| **Sensitivity** | 0.40 (0.28-0.53) | 0.40 (0.28-0.53) |
| **Specificity** | 0.57 (0.52-0.61) | 0.63 (0.59-0.68) |
| **Positive predictive value** | 0.13 (0.09-0.18) | 0.15 (0.10-0.21) |
| **Negative predictive value** | 0.86 (0.81-0.90) | 0.87 (0.83-0.91) |
| LTE: life-threatening event, NTS: Netherlands Triage Standard, OHS-PC: out-of-hours primary care, SOB: shortness of breath | | |

**Table S8. Accuracy of NTS urgency level and ‘final’ urgency allocation for detecting LTE (prevalence of 27.4%) of 506 callers aged 60-79 years who called the OHS-PC with SOB.**

|  | **NTS urgency allocation (95% CI)** | **‘Final’ urgency allocation (95% CI)** |
| --- | --- | --- |
| **Sensitivity** | 0.60 (0.51-0.68) | 0.60 (0.52-0.69) |
| **Specificity** | 0.54 (0.49-0.59) | 0.57 (0.52-0.62) |
| **Positive predictive value** | 0.33 (0.27-0.39) | 0.35 (0.29-0.41) |
| **Negative predictive value** | 0.78 (0.72-0.83) | 0.79 (0.74-0.84) |
| LTE: life-threatening event, NTS: Netherlands Triage Standard, OHS-PC: out-of-hours primary care, SOB: shortness of breath | | |

**Table S9. Accuracy of NTS urgency level and ‘final’ urgency allocation for detecting LTE (prevalence of 24.9%) of 261 callers older than 80 years who called the OHS-PC with SOB.**

|  | **NTS urgency allocation (95% CI)** | **‘Final’ urgency allocation (95% CI)** |
| --- | --- | --- |
| **Sensitivity** | 0.66 (0.53-0.77) | 0.58 (0.46-0.70) |
| **Specificity** | 0.49 (0.42-0.56) | 0.51 (0.44-0.58) |
| **Positive predictive value** | 0.30 (0.23-0.38) | 0.28 (0.21-0.37) |
| **Negative predictive value** | 0.81 (0.73-0.88) | 0.79 (0.70-0.85) |
| LTE: life-threatening event, NTS: Netherlands Triage Standard, OHS-PC: out-of-hours primary care, SOB: shortness of breath | | |
